# A longitudinal analysis of symptom burden trajectories among breast cancer patients undergoing postoperative chemotherapy and related factors: a latent class growth model

**DOI:** 10.3389/fpubh.2026.1909654

**Published:** 2026-08-26

**Authors:** Wanqin Tie, Xi Zhang, Liang Yue, Lingling Yang, Meng Niu, Jiayi Zhang

**Affiliations:** 1 General Hospital of Ningxia Medical University, Yinchuan, China; 2 Shanghai Jiao Tong University School of Medicine, Shanghai, China; 3 Ningxia Medical University School of Nursing, Yinchuan, China

**Keywords:** a latent class growth model, breast cancer, influencing factor, postoperative chemotherapy, symptom burden

## Abstract

**Background:**

Symptom burden among breast cancer patients receiving postoperative chemotherapy shows marked individual variability, yet its longitudinal trajectory has not been fully characterized.

**Aim:**

To delineate heterogeneous trajectories of symptom burden and identify their influencing factors in postoperative breast cancer patients undergoing chemotherapy.

**Methods:**

We recruited postoperative breast cancer patients receiving chemotherapy from a tertiary hospital in Ningxia, China. Participants completed a general information questionnaire and the Chinese version of the M. D. Anderson Symptom Inventory (MDASI-C) at five time points: T1 (3 days post-surgery), T2 (14 days post-surgery), T3 (1–3 days before the first chemotherapy session), T4 (7–14 days after the 4th chemotherapy session), and T5 (7–14 days after the 8th chemotherapy session). We applied latent class growth models to identify distinct symptom burden trajectories and used logistic regression to examine factors associated with trajectory membership.

**Results:**

Three distinct trajectories of symptom burden were identified: the high-risk group (*n* = 18, 8.1%), the steadily increasing group (*n* = 154, 67.3%), and the gradually improving group (*n* = 53, 24.6%). Multinomial logistic regression analysis, using the gradually improving group as the reference, indicated that employment status (OR = 0.464, 95% CI: 0.234–0.922, *p* = 0.028) and a higher level of disease-related knowledge (OR = 0.351, 95% CI: 0.161–0.764, *p* = 0.008) serve as protective factors against adverse symptom burden trajectories.

**Conclusion:**

There is notable heterogeneity within the population regarding symptom burden trajectories. In comparison to patients in the gradually improving group, those who are employed and possess a higher level of disease-related knowledge are significantly less likely to be classified within the steadily increasing group. Clinicians should prioritize the early identification of high-risk populations, engage in dynamic symptom monitoring, and implement precision management strategies based on trajectory classifications to mitigate symptom burden and enhance quality of life.

## 1 Introduction

In recent decades, breast cancer has emerged as a significant threat to women’s health in China, marked by rising incidence rates (1, 2). Surgery, in conjunction with chemotherapy, serves as the primary treatment modality, playing a vital role in enhancing survival rates and improving prognosis for breast cancer patients. However, the treatment process often subjects patients to physical discomfort and behavioral impairments resulting from surgical interventions, which impose considerable psychological and physiological burdens (3). Long-term chemotherapy not only targets tumor cells but also adversely affects healthy cells, leading to a range of negative reactions (4). These symptoms frequently manifest concurrently during both the onset and progression of the disease, presenting in clusters that further intensify the symptom burden and diminish patients’ quality of life (5).

Improving symptom management and alleviating the burden of symptoms in breast cancer patients have emerged as urgent clinical priorities. Effective symptom management depends on understanding the characteristics of symptoms, accurately identifying high-risk populations, and implementing targeted interventions. Existing studies have predominantly employed cross-sectional designs, largely neglecting the longitudinal progression and inherent heterogeneity of symptom experiences (6, 7). This static approach does not differentiate whether patients exhibit distinct patterns, such as persistent deterioration, gradual remission, or slow escalation, thereby limiting the precision of individualized interventions. In contrast to conventional growth models that assume a single average trajectory for the entire population (e.g., latent growth curve modeling), Latent Class Growth Modeling (LCGM) does not presuppose population homogeneity; rather, it adopts a data-driven approach to identify heterogeneous latent subclasses, allowing each subclass to have its own intercept and slope parameters. This capability enables a more accurate characterization of how symptom burden trajectories diverge dynamically across different treatment stages (8).

Accordingly, the present study applied LCGM to achieve two primary objectives: (1) to delineate distinct trajectory classes of symptom burden in breast cancer patients from surgery through completion of chemotherapy, and (2) to examine differences in demographic and disease-related characteristics across these trajectory subgroups, thereby providing empirical evidence to support early risk stratification and the design of targeted clinical interventions.

## 2 Methods

### 2.1 Study design and participants

This study constitutes a longitudinal follow-up survey. Data were collected at five critical time points: T1 (3 days post-surgery), T2 (14 days post-surgery), T3 (1–3 days before the first chemotherapy session), T4 (7–14 days after the 4th chemotherapy session), and T5 (7–14 days after the 8th chemotherapy session). Convenience sampling was utilized to recruit eligible participants from a tertiary hospital in Ningxia, China, between January and December 2023. All participants were sourced from the same hospital and had undergone surgical treatment followed by postoperative chemotherapy.

Inclusion criteria included: (1) a pathologically confirmed diagnosis of breast cancer with a primary modified radical mastectomy performed on one side; (2) age under 65 years; (3) TNM stage I-III; and (4) a chemotherapy regimen of AC-T. Exclusion criteria comprised: (1) concurrent severe diseases affecting vital organs such as the heart or brain; (2) patients with severe complications or recurrent/metastatic disease; and (3) patients who withdrew from the study for any reason. This study received approval from the Ethics Committee (KYLL-2022-0603), and all participants provided informed consent. The sample size was calculated to be 5 to 10 times the number of variables (9). With 22 independent variables and a projected attrition rate of 20%, the final sample size was determined to range from 138 to 275 cases.

### 2.2 Measures

#### 2.2.1 General data questionnaire

The questionnaire was developed following a comprehensive literature review and encompasses both general demographic information and clinical disease data. The general demographic information comprises age, nationality, BMI, educational level, employment status, marital status, family residence, payment method, monthly family income, have children? financial burden, primary caregiver, hobbies, bad eating habits, night sleep satisfaction, disease-related knowledge, and the levels of physical activity. The clinical disease data includes package area, pathological type, and TNM stage.

#### 2.2.2 Chinese version of the M. D. Anderson symptom inventory

The MDASI-C is a self-assessment tool designed to evaluate multiple symptoms. Developed by the Texas University Anderson Cancer Research Organization in the United States in 2000, it has gained international acceptance (10). This instrument is applicable to various treatment modalities for cancer patients. In the present study, the MDASI-C, as revised by Wang et al. (11), was employed to assess disease-related symptoms in cancer patients. The scale consists of 19 items divided into two sections. The first section measures the severity of 13 common cancer-related symptoms experienced in the past 24 h, including pain, fatigue, nausea, disturbed sleep, distress, shortness of breath, difficulty remembering, loss of appetite, drowsiness, dry mouth, sadness, vomiting, and numbness. The second section evaluates the extent to which these symptoms interfere with daily life across six dimensions: general activity, mood, work, relationships with others, walking, and enjoyment of life. This study focuses on the symptom section of Part 1. Each item is rated on a scale from 0 to 10, where 0 indicates “no symptoms” and 10 signifies “the worst a patient can imagine.” Over the five assessment time points, the Cronbach’s *α* coefficients for the MDASI-C ranged from 0.608 to 0.856, demonstrating acceptable internal consistency reliability within the current sample.

### 2.3 Data collection

In accordance with the study objectives and clinical practice, as well as the standards established by the Chinese Anti-Cancer Association Guidelines and Standards for the Diagnosis and Treatment of Breast Cancer (2021 Edition) (12), patients undergoing initial treatment with the AC-T regimen following modified radical mastectomy received a total of 8 cycles of chemotherapy, with each cycle comprising 3 consecutive weeks of treatment. To effectively capture the key clinical milestones of the adjuvant chemotherapy trajectory while minimizing patient burden during active treatment—considering that frequent assessments may exacerbate fatigue and hinder retention in this symptom-burdened population—the data collection timepoints for this study were determined as follows: T1 (3 days post-surgery), T2 (14 days post-surgery), T3 (1–3 days before the first chemotherapy session), T4 (7–14 days after the 4th chemotherapy session), and T5 (7–14 days after the 8th chemotherapy session).

A team of research group members, all of whom underwent standardized training that included uniform instruction on interview techniques, role-playing exercises, and consistency assessments, was tasked with data collection. The T1, T2, and T3 assessments were conducted through face-to-face interviews by investigators within the hospital. Before the initial interview, the study’s purpose and significance were explained to the patients. Patients and their families were informed that the survey would be conducted over five sessions, with an emphasis on the voluntary nature of participation and the harmlessness of the results. Following the acquisition of consent, an informed consent form was signed, the initial questionnaire was completed, and contact information for both the patient and the primary caregiver was recorded to facilitate follow-up. The T4 and T5 assessments were conducted via telephone or WeChat video calls with discharged patients. To ensure data quality during remote assessments, all interviewers adhered to structured telephone interview guides that included standardized probes. Patients were prompted to anchor their responses to specific recent time points (e.g., “in the past 24 h”) to minimize recall bias. For telephone follow-ups, an appointment was scheduled with the patient in advance; during the call, patients were asked to personally answer the phone whenever possible to ensure accurate recording of their symptoms. After the follow-up, the questionnaire was reviewed item by item, and any missing information was verified with the patient and completed. The completion of the questionnaire typically takes 7–10 min.

### 2.4 Statistical analysis

This study employed SPSS 22.0 and Mplus 8.3 for statistical analysis. Initially, Latent Class Growth Modeling (LCGM) was fitted using the symptom burden scores measured at five time points (T1–T5) as observed indicators. Time was coded linearly as 0, 1, 2, 3, and 4 for T1 through T5, respectively. Regarding the variance structure, we adopted the standard LCGM specification by fixing both the intercept variance (I@0) and slope variance (S@0) to zero within each class. This assumes that individuals belonging to the same latent class follow identical average trajectories, thereby focusing the model on capturing between-class heterogeneity rather than within-class individual variability. Under this specification, models with one to five classes were estimated sequentially. The fit indices for the LCGM included: (1) the Akaike Information Criterion (AIC), the Bayesian Information Criterion (BIC), and the adjusted Bayesian Information Criterion (aBIC), where smaller values indicate better model fit; (2) the entropy value, which reflects the accuracy of individual classification, with values closer to 1 indicating higher classification precision; and (3) the Log-Likelihood Ratio Test (LMR) and the Bootstrap Likelihood Ratio Test (BLRT), where a p-value less than 0.05 for either metric suggests that the corresponding K-class model is superior to the K-1-class model. Subsequently, chi-square tests were conducted to compare demographic and clinical characteristics among trajectory groups. Finally, variables that demonstrated statistical significance in univariate analyses were included in multinomial logistic regression models to identify factors associated with symptom burden trajectories. A two-sided p value <0.05 was deemed statistically significant.

## 3 Results

### 3.1 Sample characteristics

A total of 254 participants were enrolled in this study. During the follow-up survey, 13 individuals declined to participate for personal reasons, 15 cases returned to local hospitals for further treatment, and 1 case discontinued treatment. Consequently, 29 cases were lost to follow-up, resulting in 225 individuals completing the follow-up. The effective response rate of the questionnaire was 88.6%. All participants were female, with a mean age of 50.80 ± 8.07 years.

### 3.2 Identifying symptom burden trajectories

Latent class growth modeling (LCGM) was employed to identify distinct trajectories of symptom burden across five assessment points. A series of models ranging from one to five latent classes were evaluated. As the number of latent classes increased, the Akaike Information Criterion (AIC), Bayesian Information Criterion (BIC), and adjusted BIC values exhibited a consistent decline. The three-class model was selected as the optimal solution, with an entropy value of 0.844, indicating satisfactory classification quality. The average posterior probabilities for class membership were 0.976, 0.942, and 0.890 for the three classes, respectively, all exceeding the recommended threshold of 0.70, with corresponding maximum misclassification probabilities of 2.4, 5.3, and 11.0%. The Lo–Mendell–Rubin (LMR) test was significant (*p* = 0.0005) and the bootstrap likelihood ratio test (BLRT) was highly significant (*p* < 0.001), further supporting the selection of the three-class model. The results of the model fitting are presented in Table 1.

**Table 1:** The results of model fitting (*N* = 225).

Model	AIC	BIC	aBIC	Entrony	LMR	BLRT	Category probability
1	9027.836	9051.749	9029.565	—	—	—	1
2	8883.813	8917.974	8886.282	0.944	0.0001	0.0000	0.90667/0.09333
3	8789.102	8833.511	8792.312	0.844	0.0005	0.0000	0.08000/0.68444/0.23556
4	8775.993	8830.651	8779.943	0.846	0.2912	0.0000	0.08000/0.21333/0.66667/0.04000
5	8761.238	8826.144	8765.929	0.852	0.4120	0.0000	0.04889/0.05778/0.21778/0.05778/0.61778

AIC, Akaike Information Criterion; BIC, Bayesian Information Criterion; aBIC, adjusted Bayesian Information Criterion; Entropy,entropy value; BLRT, Bootstrap Likelihood Ratio Test; LMR, Lo–Mendell–Rubin adjusted Likelihood Ratio Test.

**Figure 1:**
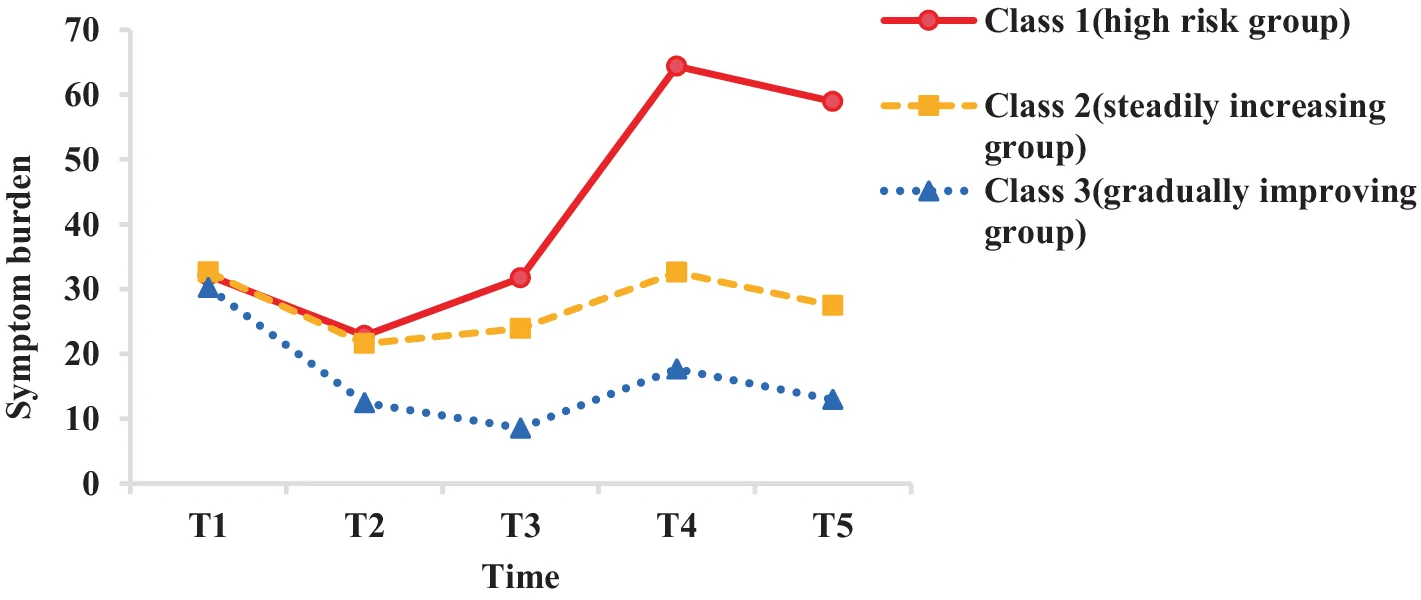
Three classes of symptom burden trajectory of patients with postoperative chemotherapy for breast cancer.

The three-class model was selected as the optimal solution. The identified trajectories are illustrated in Figure 1. The first trajectory, designated as the high-risk group (C1), included 18 patients (8.1%). Patients in this group experienced a marked and persistent increase in symptom burden throughout the follow-up period, with symptom scores rising sharply during chemotherapy. The second trajectory, termed the steadily increasing group (C2), consisted of 154 patients (67.3%). This group demonstrated a moderate but continuous increase in symptom burden over time and represented the largest subgroup in the cohort. The third trajectory, referred to as the gradually improving group (C3), comprised 53 patients (24.6%). Patients in this group exhibited an overall downward trend in symptom burden, particularly during the later stages of chemotherapy. Mean symptom burden scores across time points for the three trajectory groups are presented in Figure 1.

### 3.3 Univariate analysis of factors for symptom burden trajectories

Univariate analyses were performed to compare demographic and clinical variables across the three trajectory groups. Significant differences emerged in employment status and disease-related knowledge (*p* < 0.05) (Table 2). No significant differences were found concerning age, nationality, BMI, educational level, marital status, family residence, monthly family income, TNM stage, or pathological type (*p* > 0.05) (Table 2).

**Table 2:** Univariate analysis of symptom burden trajectory classes in breast cancer patients undergoing postoperative chemotherapy (*N* = 225).

Variables	*N* (%)	High-risk group	steadily increasing group	gradually improving group	*χ* ^2^	P
Age					3.903	0.419
≤45	47 (20.9)	2 (11.1)	35 (22.7)	10 (18.9)		
46–59	145 (64.4)	15 (83.3)	94 (61.0)	36 (67.9)		
≥60	33 (14.7)	1 (5.6)	25 (16.2)	7 (13.2)		
Nationality					2.944	0.229
Han nationality	187 (83.1)	17 (94.4)	129 (83.8)	41 (77.4)		
Other	38 (16.9)	1 (5.6)	25 (16.2)	12 (22.6)		
BMI					7.986	0.200
<18.5	8 (3.6)	1 (5.6)	4 (2.6)	3 (5.7)		
18.5–23.9	106 (47.1)	10 (55.6)	67 (43.5)	29 (54.7)		
24–27.9	86 (38.2)	7 (38.9)	61 (39.6)	18 (34.0)		
≥28	25 (11.1)	0 (0.0)	22 (14.3)	3 (5.7)		
Educational level					4.700	0.319
Primary school and below	139 (61.8)	11 (61.1)	100 (64.9)	28 (52.8)		
High school	52 (23.1)	4 (22.2)	30 (19.5)	18 (34.0)		
University and above	34 (15.1)	3 (16.7)	24 (15.6)	7 (13.2)		
Employment status					6.676	0.036
Employed	66 (29.3)	7 (38.9)	37 (24.0)	22 (41.5)		
Unemployed	159 (70.7)	11 (61.1)	117 (76.0)	31 (58.5)		
Marital status					3.918	0.121
Married	214 (95.1)	17 (94.4)	144 (93.5)	53 (100.0)		
Other	11 (4.9)	1 (5.6)	10 (6.5)	0 (0.0)		
Family residence					1.668	0.434
Urban	128 (56.9)	9 (50.0)	85 (55.2)	34 (64.2)		
Rural	97 (43.1)	9 (50.0)	69 (44.8)	19 (35.8)		
Package area					4.257	0.119
Left	105 (46.7)	11 (61.1)	75 (48.7)	19 (35.8)		
Right	120 (53.3)	7 (38.9)	79 (51.3)	34 (64.2)		
Payment method					6.626	0.157
Medical insurance	138 (61.3)	13 (72.2)	86 (55.8)	39 (73.6)		
Rural cooperative insurance	63 (28.0)	4 (22.2)	48 (31.2)	11 (20.8)		
Self-funded	24 (10.7)	1 (5.6)	20 (13.0)	3 (5.7)		
Monthly family income					2.690	0.847
<1,000	37 (16.4)	1 (5.6)	27 (17.5)	9 (17.0)		
1,000–2,999	57 (25.3)	5 (27.8)	39 (25.3)	13 (24.5)		
3,000–5,000	75 (33.3)	8 (44.4)	48 (31.2)	19 (35.8)		
>5,000	56 (24.9)	4 (22.2)	40 (26.0)	12 (22.6)		
Have children?					0.405	0.823
Yes	218 (96.9)	18 (100.0)	148 (96.1)	52 (98.1)		
No	7 (3.1)	0 (0.0)	6 (3.9)	1 (1.9)		
Financial burden					0.882	0.643
Yes	162 (72.0)	14 (77.8)	108 (70.1)	40 (75.5)		
No	63 (28.0)	4 (22.2)	46 (29.9)	13 (24.5)		
Primary caregiver					5.326	0.255
Spouse	124 (55.1)	6 (33.3)	86 (55.8)	32 (60.4)		
Children	74 (32.9)	10 (55.6)	48 (31.2)	16 (30.2)		
Other	27 (12.0)	2 (11.1)	20 (13.0)	5 (9.4)		
Disease-related knowledge					17.326	0.002
Most	54 (24.0)	9 (50.0)	25 (16.2)	20 (37.7)		
Some	67 (29.8)	4 (22.2)	50 (32.5)	13 (24.5)		
None	104 (46.2)	5 (27.8)	79 (51.3)	20 (37.7)		
The main ways to acquire knowledge					4.110	0.128
Network	108 (48.0)	6 (33.3)	71 (46.1)	31 (58.5)		
Other	117 (52.0)	12 (66.7)	83 (53.9)	22 (41.5)		
Hobbies					1.776	0.411
Yes	74 (32.9)	5 (27.8)	55 (35.7)	14 (26.4)		
No	151 (67.1)	13 (72.2)	99 (64.3)	39 (73.6)		
Bad eating habits					0.181	0.913
Yes	44 (19.6)	4 (22.2)	29 (18.8)	11 (20.8)		
No	181 (80.4)	14 (77.8)	125 (81.2)	42 (79.2)		
Night sleep satisfaction					3.535	0.473
Satisfied	77 (34.2)	7 (38.9)	52 (33.8)	18 (34.0)		
Mediocre	93 (41.3)	4 (22.2)	66 (42.9)	23 (43.4)		
Dissatisfied	55 (24.4)	7 (38.9)	36 (23.4)	12 (22.6)		
Physical activity					2.836	0.586
Often	84 (37.3)	8 (44.4)	58 (37.7)	18 (34.0)		
Occasionally	110 (48.9)	6 (33.3)	75 (48.7)	29 (54.7)		
Never	31 (13.8)	4 (22.2)	21 (13.6)	6 (11.3)		
Pathological type					4.104	0.383
Non-invasive cancer	1 (0.4)	0 (0.0)	0 (0.0)	1 (1.9)		
Invasive special cancer	12 (5.3)	0 (0.0)	9 (5.8)	3 (5.7)		
Invasive non-special cancer	212 (94.2)	18 (100.0)	145 (94.2)	49 (92.5)		
TNM stage					4.484	0.345
I	85 (37.8)	7 (38.9)	61 (39.6)	17 (32.1)		
II	96 (42.7)	10(55.6)	64 (41.6)	22 (41.5)		
III	44 (19.6)	1(5.6)	29 (18.8)	14 (26.4)		

### 3.4 Multivariate logistic regression analysis of factors influencing symptom burden trajectories

Variables demonstrating statistical significance in univariate analyses were incorporated into multinomial logistic regression models to identify independent predictors of symptom burden trajectories (Table 3). Using the gradually improving group (C3) as the reference category, the results indicated that employed patients were significantly less likely to belong to the steadily increasing group (C2) compared to unemployed patients (OR = 0.464, 95% CI: 0.234–0.922, *p* = 0.028). Likewise, patients who reported substantial disease-related knowledge were less likely to be classified in the steadily increasing group than those lacking such knowledge (OR = 0.351, 95% CI: 0.161–0.764, *p* = 0.008) (Table 3). These findings suggest that employment status and disease-related knowledge may act as protective factors against the worsening of symptom burden trajectories during postoperative chemotherapy (Table 3).

**Table 3:** Multivariable analysis of symptom burden trajectory classes in breast cancer patients undergoing postoperative chemotherapy (*N* = 225).

Dependent variable	Independent variable		B	SE	Wald *χ*^2^	P	OR	95% CI
High-risk group	Employment status	Employed	−0.170	0.567	0.090	0.764	0.844	[0.278, 2.565]
		Unemployed	—	—	—	—	—	—
	Disease-related knowledge	Most	0.613	0.647	0.898	0.343	1.846	[0.520, 6.559]
		Some	0.242	0.768	0.099	0.753	1.274	[0.283, 5.741]
		None	—	—	—	—	—	—
Steadily increasing group	Employment status	employed	−0.767	0.350	4.800	0.028	0.464	[0.234, 0.922]
		unemployed	—	—	—	—	—	—
	Disease-related knowledge	Most	−1.048	0.397	6.959	0.008	0.351	[0.161, 0.764]
		Some	0.115	0.410	0.079	0.779	1.122	[0.503, 2.505]
		None	—	—	—	—	—	—

It is important to exercise caution when interpreting the results, as the difference between the high-risk group (C1) and the gradually improving group (C3) did not achieve statistical significance (Table 3). The odds ratio estimates for the high-risk group (C1) displayed excessively wide 95% confidence intervals (e.g., OR = 1.846, 95% CI: 0.520–6.559), indicating poor stability of the effect estimates and considerable imprecision in parameter accuracy. This instability primarily arises from the extremely limited sample size allocated to this trajectory subgroup, which comprises only 18 cases. In the context of a low event count, such a small sample size makes multivariable logistic regression susceptible to model overfitting and distortion of true effect estimates. Given these concerns, any statistical inferences drawn from this subgroup should be approached with extreme caution. Consequently, the findings from this subgroup are regarded as exploratory and suggestive, necessitating confirmation through future prospective studies with larger sample sizes.

## 4 Discussion

### 4.1 Dynamic changes in symptom burden during postoperative chemotherapy

This longitudinal study demonstrated that the symptom burden among postoperative breast cancer patients undergoing chemotherapy exhibited a dynamic pattern characterized by an initial decrease, followed by an increase, and then a gradual improvement over time.

Immediately after surgery, patients experienced significant physical trauma and psychological stress, resulting in a relatively severe symptom burden. During the early postoperative recovery phase, the symptom burden temporarily decreased, likely due to the implementation of enhanced recovery after surgery (ERAS) strategies and advancements in perioperative care (13). However, the symptom burden increased again prior to and during chemotherapy, probably due to anticipatory anxiety regarding treatment outcomes and chemotherapy-related adverse effects. As chemotherapy cycles progressed, cumulative toxicities and treatment-related complications further exacerbated symptom distress. By the end of chemotherapy, the symptom burden gradually declined. This improvement may reflect patients’ psychological adaptation to treatment, symptom management support from caregivers, and relief associated with perceived disease control. Previous longitudinal studies have similarly reported fluctuations in symptoms throughout chemotherapy, indicating that symptom experiences are highly dynamic rather than static (4).

These findings underscore the necessity of continuous symptom monitoring throughout the treatment trajectory. Caregivers should proactively assess symptom changes at various treatment stages and implement timely interventions to alleviate symptom distress and enhance quality of life.

### 4.2 Heterogeneity of symptom burden trajectories

The present study identified three distinct trajectories of symptom burden among postoperative breast cancer patients undergoing chemotherapy: the high-risk group, the steadily increasing group, and the gradually improving group. These findings underscore significant interindividual variability in symptom experiences during cancer treatment. The high-risk group, comprising only 8.1% of the sample, exhibited a consistently worsening symptom burden throughout the follow-up period. Patients in this subgroup may have heightened vulnerability to treatment-related toxicities or diminished coping resources, rendering them particularly prone to severe symptom distress. Consequently, early identification and intensive symptom management for this population are of clinical importance. The steadily increasing group constituted the majority of participants. Although their symptom burden increased more gradually than that of the high-risk group, persistent symptom aggravation could still adversely impact treatment adherence and quality of life. This indicates that even patients with relatively stable symptom trajectories necessitate ongoing clinical attention. Patients in the gradually improving group demonstrated a reduction in symptom burden over time, reflecting enhanced adaptation and recovery during treatment. However, the decrease in symptom scores was relatively modest, aligning with prior longitudinal studies that indicate symptom resolution following chemotherapy completion is frequently incomplete, with significant recovery occurring primarily during the post-treatment follow-up period rather than at the cessation of treatment (14–16). Since our follow-up concluded at the end of chemotherapy, we could not capture the longer-term recovery trajectory beyond active treatment. These findings imply that, even for patients exhibiting comparatively favorable trajectories, symptom management should remain a critical aspect of supportive care throughout the entire treatment process, and post-treatment surveillance may be necessary to monitor delayed recovery.

In contrast to earlier studies that identified only high and low-symptom trajectories (17), the current findings offer a more nuanced understanding of the heterogeneity in symptom burden. Variations in study populations, treatment regimens, and statistical methodologies may account for these discrepancies. Notably, the application of LCGM in this study facilitated the identification of clinically relevant subgroups that may benefit from tailored interventions.

### 4.3 Influencing factors of symptom burden trajectories

#### 4.3.1 Employment status as a protective factor

In our cohort, employed patients were significantly less likely to belong to the group experiencing a steadily increasing symptom burden compared to unemployed patients. While this association is both biologically and psychosocially plausible, two critical methodological considerations must be acknowledged before drawing causal inferences. First, reverse causality cannot be excluded; patients with better baseline functional status may have been more capable of maintaining employment, rather than employment directly mitigating symptom deterioration. Second, employment status may reflect patients’ economic reserves and social resources; improved economic conditions are linked to higher treatment adherence, better nutritional support, and greater access to out-of-pocket medications. Even after adjusting for clinical covariates, unmeasured economic-related residual confounding may persist. Considering these limitations, the observed association may still be partially attributable to the alleviation of financial stress, the maintenance of identity and social participation, and workplace social support that buffers psychological distress (18, 19). However, previous findings have been inconsistent, and discrepancies may arise from variations in sociocultural contexts, employment protection, and healthcare payment systems. Therefore, our results should be regarded as exploratory and hypothesis-generating, and future prospective studies that incorporate multidimensional economic and resource indicators are warranted to validate these associations.

#### 4.3.2 Disease-related knowledge as a protective factor

Greater disease-related knowledge has been identified as a protective factor influencing symptom burden trajectories. Patients who possess a better understanding of breast cancer and its treatment are less likely to experience an increase in symptom burden. Those with higher levels of disease knowledge may exhibit enhanced self-management skills, improved coping strategies, and greater confidence in treatment processes. Better-informed patients are also more likely to interpret treatment-related symptoms rationally, which can reduce uncertainty, fear, and anxiety (20). Furthermore, patients with stronger educational backgrounds and proficient reading or writing skills may actively seek medical information and social support resources, thereby enhancing their participation in treatment and rehabilitation. Notably, while general education level did not achieve statistical significance in our analysis—potentially due to its limited variability within this predominantly low-to-middle education sample—disease-related knowledge represents a more specific and dynamic construct that directly influences symptom management behaviors. Collectively, these factors may contribute to improved psychological adjustment and a reduced symptom burden. These findings underscore the importance of comprehensive patient education throughout the chemotherapy process. Caregivers should implement individualized educational interventions utilizing various formats, including educational videos, peer-support programs, and digital communication platforms such as WeChat groups, to enhance patients’ understanding of disease management and bolster their self-care capacity.

## 5 Clinical implications

This study presents several practical implications for clinical care. First, trajectory-based classification facilitates a tiered model of care and education. The high-risk group may benefit from multidisciplinary support, weekly follow-ups, and targeted training on symptom self-assessment and early help-seeking, with educational materials customized to their health literacy levels. The steadily increasing group may be managed through enhanced monitoring and periodic telephone check-ins, while the gradually improving group may continue with routine care, thereby optimizing the allocation of limited healthcare resources. Second, predictors of high-risk trajectory membership—including elevated baseline symptom scores, unemployment, and limited disease-related knowledge—can inform the development of pre-chemotherapy risk screening tools. Clinicians should pay particular attention to patients exhibiting these characteristics by reinforcing pre-chemotherapy education, follow-up planning, and monitoring employment status during treatment. Additionally, integrating symptom trajectory models into electronic health applications shows promise for enabling dynamic monitoring and early warning systems, transitioning follow-up from fixed intervals to response-adaptive surveillance. Clinicians must also remain vigilant for new or worsening symptoms in breast cancer patients and conduct timely assessments when necessary. Collectively, these strategies provide a practical framework for precision-based symptom management in breast cancer patients undergoing postoperative chemotherapy.

## 6 Conclusion

Utilizing the LCGM, the symptom burden in patients receiving postoperative chemotherapy for breast cancer was categorized into three distinct subgroups, each demonstrating dynamic trends. This classification aids caregivers in comprehending the heterogeneity of symptom burden fluctuations from a developmental standpoint. Additionally, the results affirm that employment status and disease-related knowledge are significant factors affecting the categorization of symptom burden trajectories. Consequently, caregivers are encouraged to adopt dynamic symptom monitoring tailored to the specific characteristics of each trajectory group, thereby maximizing the predictive utility of trajectory classifications for targeted management, ultimately reducing symptom burden and enhancing patients’ quality of life.

## 7 Limitations and future directions

This study has several limitations. First, the single-center convenience sampling design and the intentional restriction to patients receiving the AC-T regimen—while enhancing internal validity by reducing clinical heterogeneity—may restrict the generalizability of our findings to other healthcare settings or chemotherapy protocols with varying toxicity profiles and supportive care requirements. Second, the six-month follow-up, which includes five assessment points, although capturing the major phases of postoperative chemotherapy, may not adequately reflect long-term symptom dynamics beyond the active treatment period. Third, the absence of objective clinical measures (e.g., treatment adherence or inflammatory biomarkers) and the lack of joint modeling with quality-of-life outcomes hinder a comprehensive understanding of the biological, behavioral, and functional mechanisms that underlie differences in trajectory. Furthermore, the nature of convenience sampling may introduce selection bias, as voluntarily enrolled patients might exhibit higher health literacy or adherence, potentially influencing the distribution of trajectory class estimates. Given these limitations, our findings should be regarded as exploratory. Future studies with extended follow-up, more frequent assessments, and multicenter designs are necessary to improve external validity. Additionally, incorporating multidimensional objective biological and behavioral indicators will further clarify the mechanisms through which socioeconomic and behavioral factors impact symptom burden trajectories.

## Data Availability

The raw data supporting the conclusions of this article will be made available by the authors, without undue reservation.
